# An overview of research on the association between microplastics and central nervous system disorders

**DOI:** 10.3389/fpubh.2025.1629181

**Published:** 2025-10-09

**Authors:** Xiaohua Shi, Yukai Wang, Lei Xu

**Affiliations:** Department of Neurology, China-Japan Union Hospital of Jilin University, Changchun, China

**Keywords:** microplastics, central nervous system, neurological disorders, pollution, health effects

## Abstract

As plastic pollution continues to escalate, microplastics have emerged as a major global contaminant, raising significant concerns about their potential effects on human health. In recent years, the widespread presence of microplastics has been linked to various health problems, particularly their impact on central nervous system (CNS) disorders, which are increasingly becoming a focus of scientific research. Current evidence indicates that microplastics can enter the human body through inhalation, ingestion, and skin absorption. Once they penetrate the body, these particles can accumulate in neural tissues, leading to detrimental changes such as inflammation, oxidative stress, and neuronal damage. This review aims to systematically explore the correlation between microplastic exposure and central nervous system disorders, analyze and summarize the underlying mechanisms, and provide a scientific basis for public health risk assessment and environmental policy formulation.

## 1 Introduction

Microplastics are tiny plastic particles, measuring less than 5 mm in diameter, and they are found throughout our environment, including in oceans, rivers, land, and even the atmosphere. These particles primarily originate from the breakdown of larger plastic products, industrial activities, and the everyday wear and tear of plastic items. For example, the wear of vehicle tires, the washing of textiles, and the use of personal care products significantly contribute to the release of microplastics into the environment. Larger plastic items, known as macroplastics, such as packaging, fishing nets, and tires, break down into microplastics through various processes, including photodegradation, wave erosion, biotic interactions, and mechanical abrasion. This results in secondary microplastics and even smaller particles known as nanoplastics, which are less than 1 μm in diameter. Once in the environment, microplastics and nanoplastics can enter organisms through inhalation, ingestion, and skin absorption. These particles are present in all aquatic ecosystems and can accumulate in organisms across different trophic levels, with evidence of trophic transfer observed in marine species such as fish and mammals (1). These plastic particles can also act as vectors for various chemical contaminants, such as heavy metals. Due to their minute size, nanoplastics are capable of penetrating biological membranes and causing significant biological harm (2). Microplastics have been detected in the organs of various animals, including the brains of fish and mice, raising concerns about their potential effects on the central nervous system (CNS) (2). Microplastics pose a threat to ecosystems and carry potential risks to human health, with their impact on the CNS attracting increasing attention (2). The presence of microplastics may potentially harm the central nervous system by affecting neuronal function and survival through multiple mechanisms, including neuroinflammation, oxidative stress, cytotoxicity, and hormonal disruption (3). Microplastics exert multidimensional effects on the nervous system at the molecular level [e.g., microplastic exposure interferes with the ROS/TGF-*β*/Akt/FoxO3a signaling pathway (4)], cellular level [microplastics disrupt tight junctions between endothelial cells and enter endothelial cells via endocytosis (5)], and organ level [e.g., in the hippocampus, exposure to nanopolystyrene induces P53-mediated ferritinophagy and ferroptosis in the rat hippocampus (6)]. Investigating the impact of microplastics on the central nervous system not only contributes to understanding their health risks but also provides a scientific basis for formulating relevant public health policies.

## 2 Microplastics and the central nervous system

### 2.1 Biocompatibility and bioaccumulation of microplastics

The physical and chemical properties of microplastics, such as size, shape, surface charge, and chemical composition, directly influence their environmental transport and biocompatibility, with smaller microplastic particles leading to stronger biological interactions (7). Humans are exposed to microplastics through multiple routes, including oral ingestion via the digestive system, inhalation through the respiratory system, and dermal contact. Additionally, the olfactory pathway has been identified as another potential route for microplastics to enter the brain (8), the anatomical specificity of this region may facilitate the direct translocation of microplastics from the nasal cavity to the brain, thus enabling them to circumvent the blood–brain barrier. The migration and accumulation mechanisms of microplastics in organisms are complex, primarily involving processes such as physical adsorption, cellular uptake, and biological transport (9). Microplastics can be ingested through the digestive tract, accumulate in the intestines, and subsequently disseminate to other tissues via systemic circulation (9). In this process, the size, shape, and surface properties of microplastics significantly influence their uptake efficiency and biodistribution, for instance, microplastics smaller than 1,000 μm are more readily absorbed and transported within organisms (10). Additionally, the accumulation of microplastics is modulated by the metabolic state of the organism, environmental factors (e.g., temperature and pH), and interactions with other pollutants (7). The biocompatibility and bioaccumulative characteristics of microplastics pose potential threats to human health (11).

### 2.2 Effects of microplastics on neural cells

The exposure to polystyrene nanoplastics exerted significant effects on primary neurons in mice, resulting in reduced viability of mixed neuronal cells, reactive astrogliosis, and induction of neuronal apoptosis (12). Another study reported that 28-day oral exposure to polystyrene (PS) microplastics resulted in a reduction in the number of Nissl bodies and affected the cellular architecture of neurons (13). Brain-derived neurotrophic factor (BDNF) is a key regulator of neuronal survival and synaptic plasticity,exposure to microplastics induces significant downregulation of BDNF gene expression, leading to morphological and functional impairments in prefrontal cortical neurons, which may consequently affect cognitive and emotional functions, microplastics directly disrupt neuronal function by triggering oxidative stress and cellular damage (14, 15). The study demonstrates that the uptake and toxicity of polystyrene microplastics (PS-MPs) in a blood–brain barrier (BBB) model are significantly influenced by microplastic size and inflammatory conditions (16). Polystyrene microplastics with a diameter of 0.2 μm exhibit higher uptake capacity and cellular damage compared to 1.0 μm microplastics (16). After 24-h and 72-h exposures, the permeability increased by 15.6-fold and 27.3-fold, respectively, indicating that smaller microplastics more readily cross the blood–brain barrier, leading to more severe damage. Under inflammatory conditions induced by tumor necrosis factor-*α* (TNF-α), the uptake and toxicity of microplastics were markedly enhanced, suggesting their potential risks in inflammatory states (16).

### 2.3 Impacts of microplastics on neurodevelopment

During pregnancy and lactation, the nervous system of the offspring is not fully developed and is susceptible to external environmental influences. A study (17) demonstrated that maternal exposure to PS-NPs resulted in a significant reduction in cortical thickness in fetal mice, along with disrupted neuronal migration in the cerebral cortex of the offspring, this disruption was characterized by increased proliferation of superficial neurons and a decreased number of deep-layer neurons. Additionally, synapses in the hippocampal region exhibited a significantly widened synaptic cleft and reduced postsynaptic density (17). These alterations were consistent with anxiety-like behavior and spatial memory deficits in the offspring (17). The study demonstrated that microinjection of various types of fluorescently labeled nanoparticles (NPs) into zebrafish embryos resulted in significant locomotor deficits in NP-exposed larvae (18). This effect was associated with upregulation of neurotransmitter receptors in neurons and reduced acetylcholinesterase activity, revealing the potential neurotoxic impact. The findings highlight that maternally derived nanoplastics may impair offspring neurodevelopment through reproductive transmission (18). To investigate the effects of microplastics on neurodevelopment in human pluripotent stem cell-derived cortical spheroids, it was found that long-term exposure (4 to 30 days) led to decreased cell viability and downregulated the expression of mature neuronal markers, such as *β*-tubulin III and the cortical layer VI markers TBR1/TBR2, indicating adverse effects of microplastic exposure on early human brain development. Furthermore, the study noted that smaller microplastics are more readily endocytosed by cells, while larger ones are primarily located extracellularly (19). Microplastics not only alter the biochemical properties of cells but may also cause neuronal damage and dysfunction by inducing oxidative stress and cellular stress responses (19). Polystyrene nanoplastics (PS-NPs) can transfer across the placenta into the fetal brain and accumulate significantly in multiple brain regions, including the cerebellum, hippocampus, striatum, and prefrontal cortex. This biased distribution may be related to the developmental sequence of different brain regions. Gestational exposure to PS-NPs markedly affects myelination in the cerebellum, particularly since the highest accumulation of PS-NPs occurs in the cerebellum of offspring (20). Compared to control rats, PS-NPs exposure leads to a significant reduction in the expression of myelin basic protein (MBP) and myelin oligodendrocyte glycoprotein (MOG), decreased myelin thickness, increased apoptosis, and a decline in the number of oligodendrocytes (20). These alterations ultimately result in motor function deficits (20). Maternal administration of polystyrene nanoparticles (PSNPs) during gestation and lactation alters the function of neural stem cells (NSCs), neural cell composition, and brain tissue architecture in offspring (21). *In vitro* experiments revealed that PSNPs induce molecular and functional deficits in NSCs. Offspring exposed to high concentrations of PSNPs exhibited sex-specific impairments in neurophysiological and cognitive functions, suggesting that high-dose PSNPs exposure may elevate the risk of neurodevelopmental deficits (21). PSNPs perturbed neural cell composition in the brain, particularly impairing hippocampal NSC proliferation and multi-lineage differentiation, offspring from PSNP-exposed dams displayed neurophysiological abnormalities, with female offspring showing significant cognitive deficits associated with elevated GABA levels (21). Microplastics can not only enter human cells, triggering inflammation and oxidative stress, but also interfere with normal cellular activities, ultimately affecting neurodevelopment (21). Microplastics can release toxic chemicals that may induce cytotoxicity and oxidative stress during neural development (22); the physical characteristics of microplastics, such as size and shape, may influence their distribution and biocompatibility within organisms, leading to abnormalities in neural cell development. Studies have found that zebrafish embryos exposed to polystyrene microplastics exhibited reduced locomotor activity and impaired neuronal development, which were associated with decreased expression of neurodevelopment-related genes (23); by affecting the synthesis and release of neurotransmitters, microplastics alter signal transmission between neurons, thereby interfering with the formation and function of neural networks (24). These mechanisms collectively may contribute to neurodevelopmental disorders, consequently affecting cognitive and behavioral functions. Compared to adults, infants and young children exhibit heightened vulnerability during brain and organ development. Toxic substances ingested by mothers may permeate into offspring brains through breast milk. Pregnant women should minimize exposure to potentially harmful materials such as nanoplastics to reduce adverse effects on fetal neurodevelopment.

### 2.4 Mechanistic correlations between microplastics and neurological disorders

**Figure 1:**
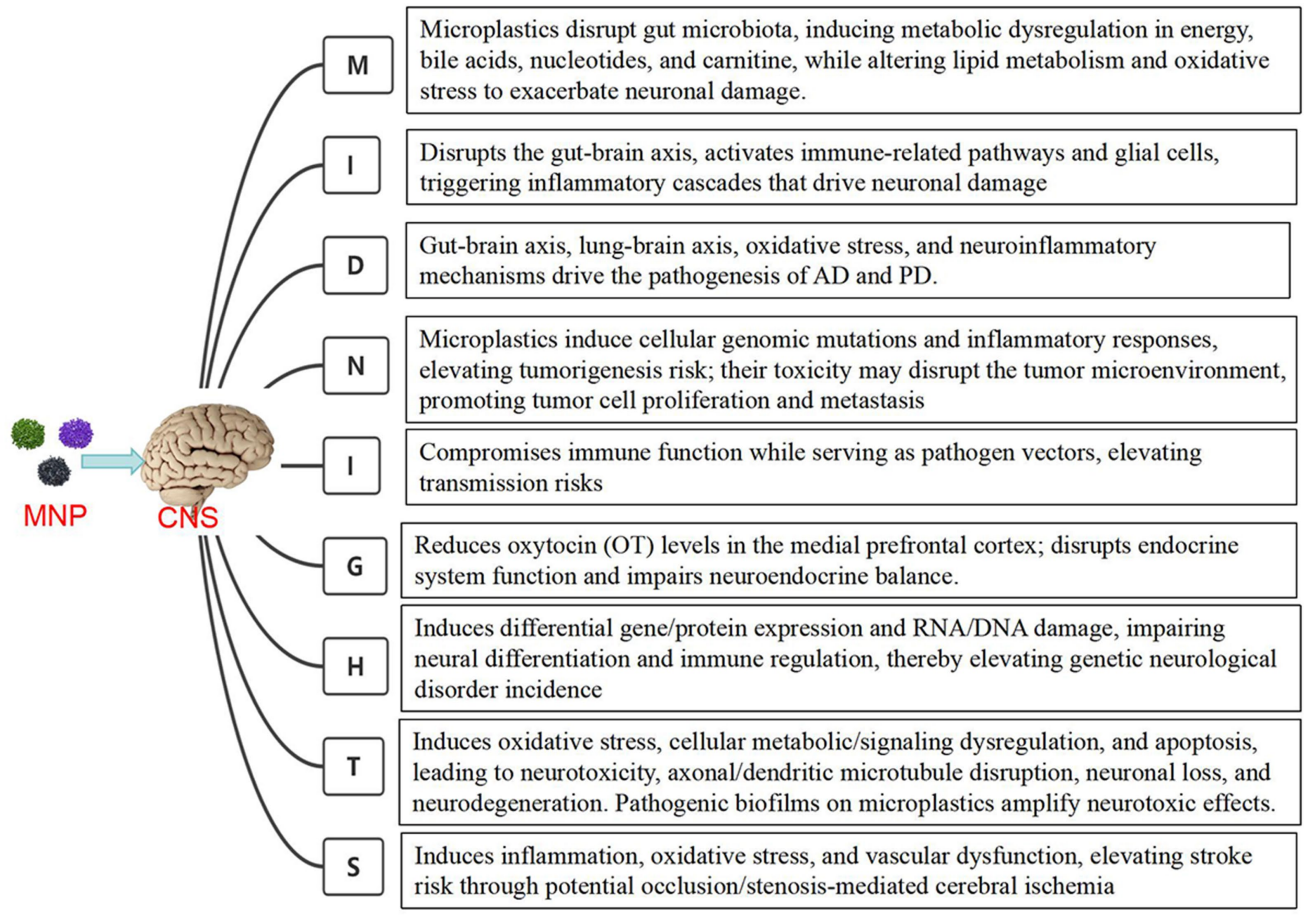
The mechanisms of microplastics associated with CNS diseases.

Neurological disorders are classified based on their localization and etiology, using the “Midnights” principle for categorization by causes. M: Metabolic disorders (e.g., central pontine myelinolysis, hypoxic–ischemic encephalopathy, hepatic encephalopathy, Wilson’s disease) and malnutrition-related conditions (e.g., subacute combined degeneration, Wernicke’s encephalopathy). I: Inflammatory/immune-mediated diseases (e.g., multiple sclerosis, neuromyelitis optica spectrum disorders, autoimmune encephalitis). D: Neurodegenerativediseases (e.g., Alzheimer’s disease, Parkinson’s disease, frontotemporal dementia, amyotrophic lateral sclerosis). N: Neoplasms (e.g., gliomas, lymphomas, medulloblastomas, pituitary adenomas). I: Infectious diseases (e.g., bacterial meningitis, herpes simplex encephalitis, neurocysticercosis). G: Endocrine disorders (e.g., hypothyroidism, hyperparathyroid encephalopathy, Addison’s syndrome). H: Hereditary disorders (e.g., hereditary ataxia, Charcot–Marie–Tooth disease, myotonic dystrophy, neurofibromatosis). T: Toxicological/traumatic injuries (e.g., chemical/neurotoxin exposure, diffuse axonal injury, spinal cord concussion). S: Stroke and cerebrovascular diseases (e.g., cerebral infarction, intracerebral hemorrhage). Exposure to microplastics may impact neurological health through multiple mechanisms, including metabolic disturbances, inflammatory responses, and neurodegenerative alterations. These mechanisms involve intracellular processes such as oxidative stress, neuroinflammation, and apoptosis, potentially leading to impaired neural function and the onset of diseases (25). Microplastic-associated mechanisms underlying CNS MIDNIGHTS-related diseases are illustrated in Figure 1.

#### 2.4.1 Association between microplastics and CNS metabolic disorders

Microplastics contribute to neuronal dysfunction by interfering with metabolic pathways,for example, microplastic exposure may lead to the development of insulin resistance and metabolic syndrome (26). Furthermore, microplastics can exacerbate neuronal damage by disrupting lipid metabolism and elevating oxidative stress, thereby promoting the occurrence of metabolic disorders. The gut microbiota is recognized as a critical regulator of gut-brain axis homeostasis. Ingestion of microplastics may induce gut microbiota dysbiosis, inflammatory cell infiltration, and impairment of the intestinal barrier function. Investigations into the effects of microplastics on the neuroendocrine system via the gut-brain axis revealed that exposure to 50 μm MPs significantly reduced colonic mucin production and induced substantial alterations in the gut microbiota, in the 50 μm - 100 μg/L exposure group, a marked decrease in oxytocin (OT) content in the medial prefrontal cortex was observed, along with damage to the blood–brain barrier, these findings highlight the potential adverse effects of environmental microplastic exposure on the neuroendocrine framework in social mammals, including humans (27). Another study demonstrated that both 200 nm and 800 nm MPs affected the gut microbiota in mice and induced metabolic disturbances in energy metabolism, bile acid metabolism, nucleotide metabolism, and carnitine metabolism, ultimately leading to neurotoxicity and cognitive impairment (28).

#### 2.4.2 Microplastics and CNS inflammatory disorders

A study (29) found that 44 nm polystyrene nanoplastics (PS-NPs) affected the brain-gut-microbiota axis and embryo-larval development of zebrafish (*Danio rerio*). Exposure to 1, 10, and 100 μg/L PS-NPs for 30 days inhibited growth and exerted adverse effects on inflammatory responses and intestinal permeability. Targeted metabolomic analysis revealed alterations in 42 metabolites involved in neurotransmission. Gene expression analysis demonstrated significant changes in the expression of inflammation- and permeability-related genes in the zebrafish intestine under different PS-NP concentrations. At low concentrations (1 and 10 μg/L), intestinal inflammation-related genes such as tumor necrosis factor *α* (TNFα) and interleukin (IL-1β) were significantly downregulated, whereas their upregulation was observed at high concentrations (100 μg/L), indicating a pronounced inflammatory response. These results suggest that PS-NP exposure disrupts regulatory mechanisms within the brain-gut-microbiota axis by altering intestinal barrier integrity and microbiota composition (29). Furthermore, *in vitro* studies have also demonstrated that microplastics induce more severe inflammatory responses in human microglial cells (HMC-3), leading to neuronal damage characterized by reduced cell body size and increased apoptosis (30). Microplastics can activate glial cells and trigger inflammatory reactions, thereby causing damage to neural cells (31). This inflammatory response not only affects the local neural environment but may also influence systemic immune reactions through the release of cytokines and chemical mediators.

#### 2.4.3 Association between microplastics and neurodegenerative disorders

Microplastics can promote neuronal apoptosis and dysfunction by inducing oxidative stress and neuroinflammation (25),the bioaccumulation of microplastics leads to protein aggregation within neurons, further exacerbating the neurodegenerative process.After oral administration, polystyrene nanoplastics (PS-NPs) can cross the blood-brain barrier (BBB) and distribute in regions such as the cortex, hippocampus, substantia nigra pars compacta (SNc), and striatum of mouse brains. Further transcriptomic analysis revealed that PS-NPs exposure significantly downregulated signaling pathways associated with neurodegenerative diseases, with the Parkinson's disease (PD) pathway being the most notably affected (32). This exposure also disrupted various cell-specific responses, including energy metabolism, mitochondrial function, and proteostasis, particularly in excitatory neurons, manifesting as reduced motor function and neuronal loss, PS-NPs exposure decreased ATP content and reduced the expression of ATP-related genes and proteins in the substantia nigra and striatum of mice, demonstrating a close relationship between energy metabolism disorders and neurodegeneration (32), Polystyrene nanoplastics (PS-NPs) can induce Parkinson's disease-like neurodegeneration in mice through energy metabolism dysfunction.

Study (33) indicates that intestinal epithelial-specific Nrf2 deficiency exacerbates polystyrene nanoplastics (PS-NPs)-induced Parkinson’s disease (PD)-like neurodegeneration. Histological analysis revealed a significant reduction in the number of Nissl bodies and dopaminergic neurons in the substantia nigra and striatum of Nrf2-deficient mice, suggesting more severe neuronal damage and death. The study also observed significant alterations in mitochondrial structure in Nrf2fl/fl-VilCre^+^ mice following PS-NP exposure, characterized by decreased cristae density and sparse matrix within mitochondria. Microplastics may contribute to intestinal vulnerability by inducing gut microbiota dysbiosis and elevating levels of the pro-inflammatory cytokine interleukin-17C (IL-17C). Such intestinal vulnerability may further aggravate PD-like neurodegeneration by enhancing neurotoxic effects (33).

Study has found that low-dose microplastic exposure accelerates dopamine neuron degeneration and induces motor deficits, the mechanism involves disruption of the intestinal mucosal barrier, immune barrier, and microbial barrier, leading to neuropathological changes resembling those of Parkinson’s disease, non-penetrating microplastics exacerbate neuroinflammation by triggering excessive reactive oxygen species generation and sustained unfolded protein response (34). Regardless of barrier penetration, in vivo exposure to microplastics plays a significant role in dopamine neuron degeneration, representing a non-independent risk factor that cannot be overlooked in PD pathogenesis (34).Both in vivo and in vitro experiments demonstrated that Western blotting and immunofluorescence revealed PS-NPs induced pyroptosis, disrupted autophagic flux, and reduced the levels of proteins involved in autophagosome-lysosome fusion. Polystyrene nanoparticles (PS-NPs) exacerbated behavioral abnormalities and caused dopaminergic neuron loss (35).According to Braak’s hypothesis, the pathological features of Parkinson’s disease are mediated by the transmission of *α*-synuclein (αS) via the gut–brain axis. Polystyrene nanoplastics accelerated the amyloid aggregation of the A53T mutant, leading to increased production of glial activation biomarkers, cytokines, and reactive oxygen species *in vitro*, as well as disruption of mitochondrial and lysosomal membrane integrity (36). These changes further altered cellular metabolic profiles associated with Parkinson’s disease pathophysiology. *In vivo*, co-administration of polystyrene nanoplastics and the A53T mutant promoted synergistic gut-to-brain transmission in mice, resulting in progressive impairment of physical and motor functions resembling typical Parkinsonian symptoms. This study (36) revealed the intervention mechanism of polystyrene nanoplastics in the gut–brain axis in Parkinson’s disease. The study employed the polymorphic oligomerization of NACore, a surrogate of *α*-synuclein relevant to Parkinson’s pathogenesis,data showed that the rate and extent of NACore oligomer formation were modulated by exposure to polystyrene nanoplastics, cellular experiments further indicated that nanoplastics enhanced the toxicity of NACore in microglial cells,simulation studies confirmed that hydrophobic interactions facilitated the binding between nanoplastics and NACore (37). These findings were validated in zebrafish embryos exposed to nanoplastics, which exhibited hatching impediments, reduced survival, and developmental abnormalities. This study (37) elucidated the role of nanoplastics in stimulating the amyloidosis of NACore derived from *α*-synuclein in Parkinson’s disease.

Microplastics and nanoplastics can be ingested by organisms through various pathways, cross the blood–brain barrier, and enter the brain, leading to oxidative stress, inhibition of acetylcholinesterase activity, alterations in neurotransmitter levels, and a series of behavioral changes (38). Adult hippocampal neurogenesis is a critical component of brain plasticity, cognitive processing, emotion, learning, and memory. Studies have found (39) that cationic nanoplastics (PS–NH3^+^) exhibit significant neurotoxicity toward neural precursor cells (NPCs), causing mitochondrial dysfunction and cell cycle arrest, thereby impairing hippocampal neurogenesis. The study revealed that PS–NH3^+^ treatment increases intracellular reactive oxygen species (ROS) levels, leading to DNA damage and G1 phase cell cycle arrest, while also disrupting mitochondrial energy metabolism and slowing cell growth. *In vivo* experiments demonstrated that prolonged exposure to PS–NH3^+^ compromises hippocampal neurogenesis and memory retention in mice. The research highlights that nanoplastics deplete the neural stem cell pool in the brain by impairing mitochondrial function and inducing cellular senescence, ultimately affecting neurogenesis in the hippocampal region and related cognitive functions, suggesting a close association between microplastics and the development of Alzheimer’s disease (39).

Another study (40) investigated the impact of microplastics on the cognitive abilities of bees. Using three-dimensional imaging technology, it was confirmed that microplastic particles with diameters of 1 to 5 micrometers could penetrate the blood–brain barrier of bees and accumulate in their brains within just 3 days, potentially causing mechanical, cellular, and biochemical damage to the central nervous system of bees. This exposure significantly reduced the bees’ responsiveness to sucrose and impaired their learning and memory abilities.

The gut-brain axis is a bidirectional neuroendocrine signaling pathway connecting the central nervous system and the intestines, with gut microbiota playing a critical role in this process. Exposure to MNPs may lead to gut microbiota dysbiosis, thereby affecting normal brain function, particularly in neurotransmitter metabolism. Studies (41) have revealed significant neurodegenerative effects of micro- and nanoplastics (MNPs) on the brain, primarily through their ability to cross the blood–brain barrier (BBB) and induce neuronal damage, they can also disrupt the balance of gut microbiota and compromise intestinal barrier integrity, further facilitating BBB penetration. MNPs accumulate in the mouse brain, resulting in a reduction in Purkinje cells and neuronal injury. Furthermore, MNP exposure is closely associated with mitochondrial dysfunction, disrupted energy metabolism, and altered neurotransmitter levels, particularly affecting cholinergic and dopaminergic neurons (41), more importantly, MNP exposure is linked to cognitive deficits, anxiety-like behaviors, and other neurobehavioral impairments.

Oral ingestion of nanoplastics (NPs) triggers the activation and reprogramming of intestinal macrophages, leading to the release of IL-1, which impacts cerebral immunity and interferes with the brain-gut axis, ultimately resulting in declined cognitive ability and short-term memory in mice (42). Cessation of NP exposure or blockade of IL-1 signaling can alleviate these NP-induced behavioral impairments (42). Ingestion of PS-MPs leads to a reduction in the intestinal mucus layer and dysbiosis of the gut microbiome, these changes are correlated with alterations in social behavior via the gut-brain axis, further analysis indicates that blocking the vagus nerve pathway improves social deficits, suggesting a key role of the gut-brain axis in mediating the neurobehavioral effects of microplastics (27).

**Figure 2:**
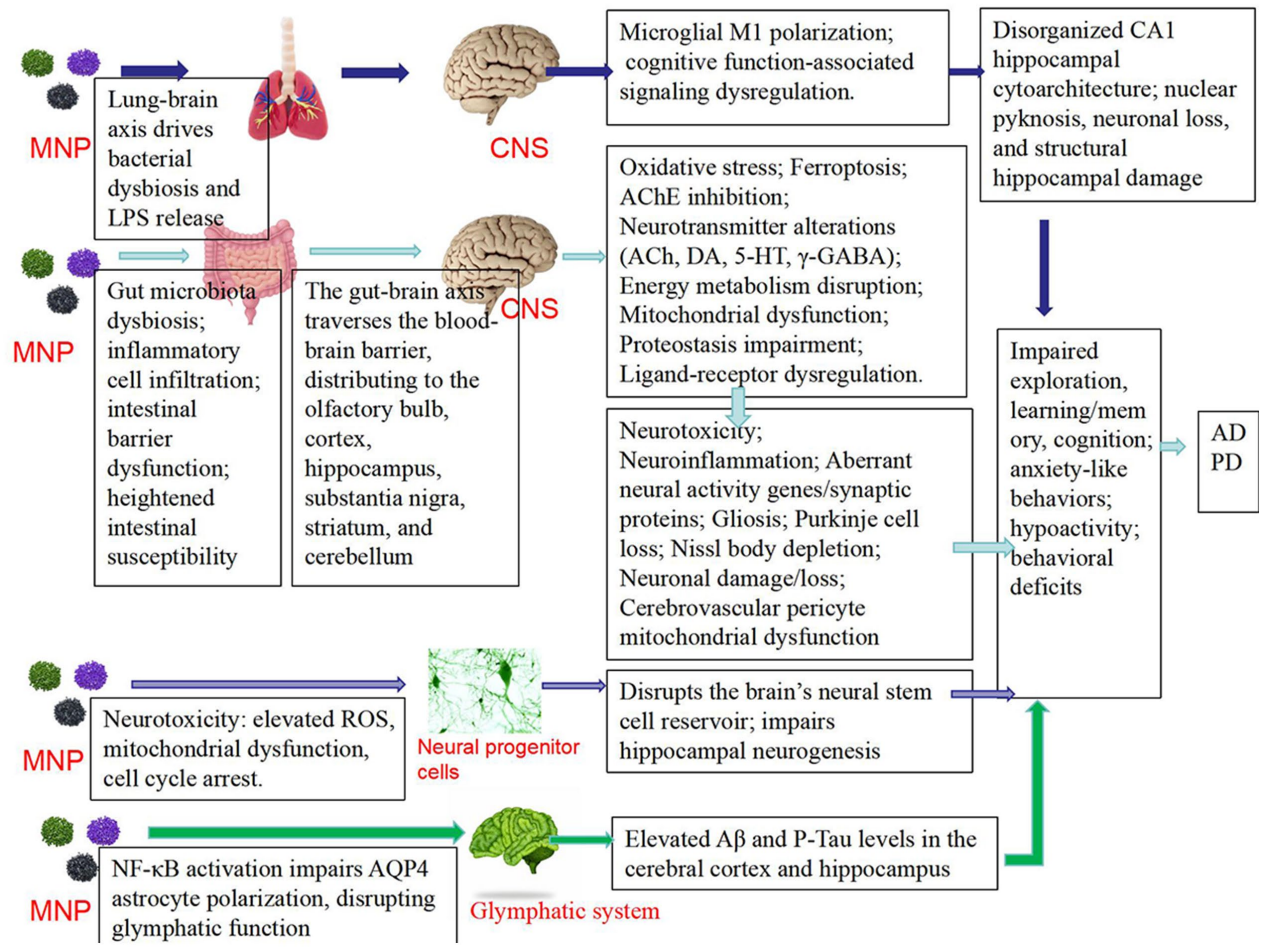
The mechanisms related to microplastics and neurodegenerative diseases such as Parkinson’s disease (PD) and Alzheimer’s disease (AD).

Zhang et al. (43) found that combined exposure to nanoplastics (NPs) and arsenic (As) resulted in decreased motor ability, increased anxiety, and impaired short-term memory. This was mediated through disruption of microbiota-gut-brain axis homeostasis, specifically manifested as reduced production of serotonin (5-HT) in the gut, leading to lower levels transported to the brain via the bloodstream, ultimately contributing to neurobehavioral deficits. These findings suggest that microplastics may exacerbate or trigger neurodegenerative diseases by interfering with microbiota-gut-brain axis homeostasis (43). Long-term oral exposure to PS-MPs causes significant deficits in learning and memory behaviors in mice (44), PS-MPs accumulate in the hippocampal region, affecting the expression of genes related to neural activity and synaptic proteins, inducing neuroinflammation, reducing the expression of immediate early genes, and altering synaptic protein profiles, ultimately leading to impaired learning and memory (44).In study on zebrafish (Symphysodon aequifasciatus) (45), micro- and nanoplastics were found to induce neurobehavioral toxicity through the brain-gut-microbiota axis. Microplastics (MFs) inhibited growth performance, while nanoplastics (NPs) reduced swimming and predation capabilities. Additionally, increased acetylcholinesterase (AChE) activity and inhibited butyrylcholinesterase (BuChE) activity were observed in the brain after plastic exposure (45). The concentrations of neurotransmitters such as acetylcholine, dopamine, and GABA increased in the brain but decreased in the gut, regarding gut microbiota, MFs exposure increased microbial richness, whereas NPs reduced the abundance of certain microbial groups. Brain transcriptome analysis revealed changes in the expression of multiple genes related to neural activity, with both MFs and NPs groups showing enrichment in pathways associated with neuroactive ligand-receptor interactions and serotonin synapse (45). These results demonstrate that microplastics induce behavioral toxicity in fish through the brain-gut-microbiota axis. The correlation between the gut-brain axis and Alzheimer’s disease has been confirmed, and Sodium Oligomannate Capsules, a drug targeting the gut-brain axis for Alzheimer’s treatment, has been clinically applied in China. Therefore, microplastic-induced disruption of gut microbiota may potentially initiate or exacerbate Alzheimer’s disease (Figure 2).

The concept of the lung–brain axis has gained increasing attention. Inhalation of polystyrene microplastics (PS-MPs) was found to induce significant negative effects on cognitive function in mice. Histological analysis revealed disorganized cell arrangement in the hippocampal CA1 region, partial nuclear condensation, and neuronal loss. The extent of damage worsened with increasing concentrations of PS-MPs. It is suggested that PS-MPs may impair cognitive function by damaging the hippocampal structure. Behavioral tests and histopathological examinations indicated injuries in both lung and brain tissues, implying that microplastics may affect neurological function indirectly via pulmonary impairment. Furthermore, RNA sequencing analysis demonstrated significant alterations in cognitive-related signaling pathways in both lung and hippocampal tissues following PS-MPs exposure, particularly affecting the expression of factors associated with neuroplasticity. Pulmonary inflammatory responses and cellular damage may indirectly contribute to neural dysfunction by promoting microbial dysbiosis and the release of endotoxins such as LPS. These findings suggest that lung injury and microbiota disruption induced by PS-MPs may lead to increased LPS levels, subsequently triggering M1 polarization of microglia and resulting in neurotoxicity. Together, these results indicate that PS-MPs affect neurological function via the lung–brain axis, representing an important mechanism underlying PS-MPs-induced cognitive impairment (46).

Microplastics can cross the blood–brain barrier, impairing the functions of neuroinflammatory secretion, transporter proteins, and receptor markers, thereby causing neurological damage and subsequently triggering various brain disorders (47). Polystyrene nanoparticles (PS-NPs) can traverse the blood–brain barrier via nasal inhalation and deposit in the brains of mice, primarily accumulating in regions such as the olfactory bulb, cerebral cortex, and cerebellum, leading to neurotoxicity and behavioral alterations (48). Smaller nanoparticles (e.g., 80 nm) exhibit higher neuronal uptake efficiency and stronger neurotoxicity compared to larger nanoparticles. Mice exposed to nanoplastics showed reduced locomotor activity and increased anxiety-like behaviors in behavioral tests. Additionally, a significant decrease in acetylcholinesterase (AChE) activity was observed in these mice, suggesting that nanoplastics may directly enter the brain via the olfactory nerve pathway, triggering inflammatory responses and neurotoxicity, ultimately impairing cognitive function (48).

The accumulation of polystyrene nanoplastics leads to increased levels of amyloid-*β* (Aβ) and phosphorylated Tau protein (P-Tau) in the cerebral cortex and hippocampus, which are metabolic waste products associated with neurobehavioral disorders and neurodegenerative diseases such as Alzheimer’s disease. PS-NH2 (aminated polystyrene nanoplastics) causes significant dose-dependent impairments in exploratory behavior and spatial learning in mice (49). PS-NH2 exposure activates the NF-κB pathway, resulting in impaired polarization of aquaporin-4 (AQP4) at astrocytic endfeet, thereby disrupting glymphatic system function. This leads to reduced clearance of cerebral Aβ and P-Tau, ultimately contributing to neurotoxicity and learning-memory deficits (49).

Human brain vascular pericytes, a critical component of the blood–brain barrier, are responsible for maintaining its integrity and clearing toxins. Plastic particles can penetrate the blood–brain barrier and potentially impair pericyte function. Exposure to microplastics leads to decreased mitochondrial function, induces oxidative stress and ferroptosis, and causes abnormal expression of genes related to mitochondrial function, such as increased expression of mitochondrial transcription factor A (TFAM), thereby associating them with neurodegenerative diseases including Alzheimer’s disease and Parkinson’s disease (50).

By inducing oxidative stress and suppressing the expression of the atoh1a gene, researchers analyzed the interaction between PS nanoplastics and the neural protein phosphoglycerate dehydrogenase (PHGDH), finding that strong binding of PS nanoplastics to PHGDH may promote the aggregation and expression of this protein, potentially leading to elevated dopamine (DA) levels. Exposure to PS nanoplastics resulted in significantly enhanced locomotor activity in zebrafish larvae, accompanied by increased DA levels and elevated acetylcholinesterase (AChE) activity. The neurotoxic mechanisms induced by PS nanoplastics contrast with typical Parkinson’s disease (PD)-like effects (51).

Exposure to PS-MPs disrupts the blood–brain barrier, causing brain tissue damage, particularly a reduction in Purkinje cells in the cerebellum, indicating impaired neural transmission (52). Significantly decreased expression levels of Occludin, Claudin 3, and ZO-1 proteins in cerebellar tissue further confirmed blood–brain barrier disruption. PS-MPs interfere with the balance of the antioxidant system via the Nrf2-Keap1-HO-1/NQO1 axis, leading to altered antioxidant enzyme activity, increased lipid peroxidation, and induction of autophagy-dependent ferroptosis in cerebellar tissue. Morphological changes in cell mitochondria, along with alterations in the expression of related proteins and genes, demonstrate that PS-MPs exposure causes severe cellular damage and apoptosis in cerebellar tissue (52).

Upon exposure to polystyrene nanoparticles (PS), cells exhibit endogenous oxidative stress, leading to the aggregation of TAR DNA-binding protein 43 (TDP-43) and subsequently triggering ALS-like pathological features. The aberrant cytoplasmic accumulation of TDP-43 facilitates the formation of PS–TDP-43 complexes, markedly enhancing the process of protein condensation and stabilization. These findings reveal that nanoscale polystyrene plastics (PS) can induce clinical manifestations resembling amyotrophic lateral sclerosis (ALS) and elucidate a distinct toxicological mechanism through which polystyrene nanoparticles contribute to ALS pathogenesis (53).

A cross-sectional study aimed to evaluate the impact of urinary microplastics (MPs) exposure on cognitive development in 5670 primary school children (ages 7–10) from Shenyang, China. Total microplastic exposure was associated with increased inattentiveness. Regression analysis revealed a dose-dependent negative association between urinary microplastic levels and cognitive development, with higher exposure levels linked to impaired working memory in both the Two-Back and Three-Back tasks. These results highlight the potential harmful effects of microplastic exposure on cognitive outcomes in children (54).

Polyethylene terephthalate (PET) nanoparticles accelerate amyloid fibril formation and alter the secondary structure of Aβ fibrils, these findings further suggest that accumulation of PET nanoparticles in the brain promotes the progression of multiple neurodegenerative diseases, including Alzheimer’s disease (55).

Transmission electron microscopy and molecular dynamics simulations demonstrated that polystyrene nanoplastics accelerate Aβ aggregation through the formation of protein coronas, facilitating peptide fibrillization via hydrogen bonding and *π*–π interactions, flow cytometry and endocytosis inhibition assays revealed that polystyrene nanoplastics impair microglial uptake of Aβ while enhancing their own cellular internalization, leading to microglial energy exhaustion and allowing Aβ aggregates to escape immune clearance. Furthermore, proteomic analysis indicated that polystyrene nanoplastics disrupt microglial homeostasis, exacerbate neuroinflammation and metabolic dysregulation, and impair ABC transporter signaling pathways crucial for Aβ clearance (56). These findings suggest that nanoplastics promote AD pathogenesis by impeding Aβ clearance and compromising neuroimmune defenses (56).

Using APP/PS1 transgenic mouse models and BV2 microglial cell systems, Yu et al. (57) systematically evaluated the effects of nanoplastic exposure on cognitive function and Alzheimer’s disease (AD) pathology, the study demonstrated that exposure to environmentally relevant doses of nanoplastics aggravates cognitive impairment and promotes *β*-amyloid (Aβ) plaque deposition. Additionally, nanoplastics were found to induce lysosomal damage and trigger microglial pyroptosis, resulting in impaired phagocytic function and reduced capacity to clear Aβ plaques (57).

Microplastics have been reviewed to induce neurodegeneration primarily through four mechanisms: exacerbating oxidative stress via reactive oxygen species (ROS) generation; triggering neuroinflammation through microglial activation and chronic inflammatory responses; inducing neurotoxicity by transporting persistent organic pollutants (POPs) and heavy metals; and accelerating *β*-amyloid pathology by enhancing the nucleation of Aβ40 and Aβ42 (58).

#### 2.4.4 The association between microplastics and central nervous system neoplasms

Toxicological testing and metabolic profiling results demonstrate that nano/micron polystyrene materials (n/mPS) exert significant toxic effects on SH-SY5Y human neuroblastoma cells, with enhanced toxicity under oxidative conditions ultimately leading to cytotoxicity and metabolic disturbances (59), revealing microplastics’ intervention in tumor cells. Microplastics increase tumorigenesis risk by inducing cellular genetic mutations and inflammatory responses (26), particularly in children and adolescents, microplastic exposure shows close correlation with brain tumor development, the toxicity of microplastics may affect the tumor microenvironment, promoting tumor cell proliferation and metastasis.

#### 2.4.5 Association between microplastics and central nervous system infectious diseases

Microplastics not only impair growth and immune function in fish but also act as vectors for pathogens, increasing the risk of pathogen transmission. A study (60) found that polystyrene nanoplastics (PS-NPs) induced significant toxic effects on brain tissue cells in orange-spotted groupers (*Epinephelus coioides*), the mechanism involves significant downregulation of immune-related genes following PS-NP exposure, which accelerates viral replication and reduces cellular and tissue resistance to viral infection. Thus, microplastic exposure can lead to functional impairment of immune cells, weaken the host’s resistance to pathogens, and increase the risk of central nervous system (CNS) infections (25). Another study analyzed cerebrospinal fluid (CSF) samples from 28 patients, divided into groups based on the presence or absence of CNS infection, the results (61) showed that concentrations of polypropylene (PP) and polyethylene (PE) were positively correlated with the CSF albumin index, suggesting that blood–brain barrier impairment may facilitate the entry of these MNPs. However, although significant increases in inflammatory markers (such as IL-6 and IL-8) were observed in patients with CNS infections, no significant association was found between MNP accumulation and the levels of these inflammatory markers. This indicates that MNP accumulation does not significantly induce or exacerbate inflammatory responses in the central nervous system.

#### 2.4.6 Association between microplastics and neuroendocrine functions in the central nervous system

In mammals, the oxytocin (OT) system is closely linked to social behavior and is sensitive to environmental stress. Previous studies have indicated that environmental toxins may influence social behavior and induce certain psychological states. One study (27) investigated the effects of microplastics on behavioral neuroendocrinology via the gut–brain axis, particularly focusing on the impact of microplastics on oxytocin levels. The results demonstrated that prolonged exposure to polystyrene microplastics (PS-MPs) significantly reduced sociability in mice and decreased oxytocin levels in the medial prefrontal cortex (mPFC). These effects were closely associated with gut microbiota dysbiosis and a reduction in the intestinal mucus layer. By exposing mice to PS-MPs of varying sizes and concentrations, it was found that larger microplastics notably impaired social behavior, revealing the potential harm of microplastics to the neuroendocrine system through interactions with gut microbiota and oxytocin signaling (27). Another study suggested that certain chemical components in microplastics can mimic endocrine hormones, disrupt hormone synthesis and release, disturb neuroendocrine balance, interfere with neural development and function, and consequently contribute to the onset of a range of neurological disorders (26).

#### 2.4.7 Microplastics and genetic disorders of the central nervous system

The study (62) demonstrated that micro/nanoplastics (MNPs) act as carriers for 4-methylbenzylidene camphor (4-MBC), exacerbating its neurotoxic and reproductive toxicity in zebrafish, at the genetic and protein expression levels, 11,736 and 4,363 differentially expressed genes (DEGs) were identified in the brain tissues of female and male zebrafish, respectively. These DEGs were associated with neural differentiation in the central nervous system and regulation of immune system processes, molecular-level investigations revealed significant impacts of co-exposure to 4-MBC and PS-NPs on the transcriptome and proteome of zebrafish brains, involving multiple biological processes such as neuronal differentiation, immune regulation, and reproduction (62). Another study (31) found that polystyrene nanoparticles (PS NPs) exposure significantly increased reactive oxygen species (ROS) levels and DNA damage in the prefrontal cortex (PFC) of mice. Comparative transcriptomic analysis between the 0 and 50 mg/kg PS NPs groups identified 987 differentially expressed mRNAs, 29 miRNAs, and 67 circRNAs (31). Functional enrichment analysis indicated that PS NPs caused substantial synaptic dysfunction, with 96 mRNAs linked to synaptic impairment, a competing endogenous RNA (ceRNA) network was constructed, comprising 27 circRNAs, 19 miRNAs, and 35 synaptic dysfunction-related mRNAs. These findings suggest that microplastics induce neurogenetic disorders by damaging various RNA and DNA molecules, thereby interfering with genetic information (31). Thus, microplastics may contribute to the increased incidence of central nervous system genetic disorders by disrupting gene and protein expression.

#### 2.4.8 Association between microplastics and toxic encephalopathy of the central nervous system

MNPs can adsorb and release multiple toxic chemicals, posing threats to ecological environments and human health. Studies have indicated that MNPs are ubiquitously present in most human samples, demonstrating a particular ability to penetrate the placenta, blood–brain barrier, and blood-testis barrier. The penetrative capacity of MNPs is influenced by various factors, including their physicochemical properties, exposure dose, route of exposure, and duration. Certain MNPs can cross the blood–brain barrier and accumulate in the mouse brain, potentially leading to neurotoxicity and cognitive dysfunction (31). Additional research has confirmed that MNPs penetrate the mouse brain and accumulate within neurons, inducing oxidative stress, cytotoxicity, and neurodegeneration. Direct exposure to MPs causes morphological alterations in human cortical and nociceptive neurons, manifested as disruption or loss of microtubule structures in axons and dendrites, accompanied by a significant reduction in neuronal length and number. Cortical neurons were observed to be more susceptible to these effects than nociceptive neurons (63). Furthermore, pathogenic bacterial biofilms attached to microplastic surfaces markedly exacerbate neurotoxic effects, underscoring the potential risks environmental microplastics pose to the nervous system. These toxic substances induce toxic neurological damage by disrupting cellular metabolism and signaling pathways, leading to oxidative stress and apoptosis.

#### 2.4.9 Association between microplastics and stroke

Current evidence indicates that microplastics may increase the risk of stroke occurrence by inducing inflammatory responses and oxidative stress (25). Long-term low-dose oral administration of PS-NPs induces excessive generation of reactive oxygen species (ROS), which on one hand triggers an inflammatory response via the NF-κB signaling pathway, and on the other hand promotes the expression of the antioxidant gene HO-1 through Nrf2. Activation of the NF-κB p65 signaling pathway leads to the phosphorylation of ERK and p38 MAPK. The activation of the MAPK signaling pathway results in a significant increase in IRS1 phosphorylation, and this hyperphosphorylation of IRS1 downregulates p-Akt expression, thereby relieving the inhibition of the key gluconeogenic gene FoxO1. With the assistance of the co-transcriptional activator PGC-1α, FoxO1 enhances the expression of the key gluconeogenic enzymes G6Pc and PEPCK, consequently increasing hepatic glycogen production. Furthermore, activated ERK promotes hepatic lipid accumulation through the ERK–PPARγ signaling cascade, while also upregulating the expression of the lipogenic enzyme ACC-1, leading to excessive accumulation of triglycerides and lipid droplets in the liver. Comprehensive results demonstrate that polystyrene nanoparticles (PS-NPs) disrupt glucose and lipid metabolism, induce insulin resistance (IR) resulting in elevated plasma glucose levels, and promote hepatic glycogen synthesis (64). Blood lipids and blood glucose are established major risk factors for stroke, while microplastics represent an emerging potential risk factor.

Recent clinical studies have demonstrated the presence of micro- and nanoplastics (MNPs) within carotid artery plaques, with these patients exhibiting a higher propensity for vascular events, one study (31) revealed through histological analysis that microplastics can directly induce vascular occlusion or stenosis, consequently leading to stroke. Although polystyrene (PS) microplastics do not promote angiogenesis, they exacerbate retinopathy by inducing apoptosis of endothelial cells and pericytes and increasing vascular leakage (65), suggesting that exposure to PS microplastics can lead to vascular dysfunction, thereby increasing the risk of stroke. The study found that microplastics (MPs) in systemic circulation can be phagocytosed by cells and induce capillary occlusion in the cerebellar cortex, these thromboembolic occlusions reduce blood flow and trigger neurological dysfunction in mice, the mechanism reveals that MPs indirectly impair tissue function by mediating cellular occlusion and disrupting local microcirculation, rather than directly penetrating tissues (66). Compared to the global cerebral ischemia control group, the accumulation of microplastics in the global cerebral ischemia + microplastics group significantly increased neuroinflammation, disruption of microtubule structure, and neuronal cell death, the study demonstrates that the accumulation of microplastics in daily life exacerbates severe neuronal cell death following cerebral infarction (67).

## 3 How to mitigate the impact of microplastics on the CNS

The study (68) investigated the role of vitamin D in mitigating the neurotoxicity and immunotoxicity induced by polystyrene nanoplastics (PS-NPs) on the brain-gut axis of zebrafish (*Danio rerio*). Transmission electron microscopy (TEM) results revealed that PS-NPs accumulated in the brain and intestine of zebrafish, leading to damage of the basement membrane of the blood–brain barrier, vacuolation of intestinal goblet cells and mitochondria. High concentrations of vitamin D reduced PS-NPs accumulation in zebrafish brain tissue by 20% and in intestinal tissue by 58.8 and 52.2%, respectively, alleviating the pathological damage caused by PS-NPs. Adequate vitamin D significantly increased serotonin (5-HT) levels and reduced anxiety-like behaviors in zebrafish induced by PS-NPs exposure. Vitamin D was found to mitigate the neurotoxicity and immunotoxicity caused by PS-NPs exposure by directionally modulating the gut virome. These findings highlight the potential of vitamin D in alleviating brain-gut-virome dysregulation induced by PS-NPs exposure and propose a potential therapeutic strategy involving dietary supplementation with sufficient vitamin D (68). RNA sequencing and bioinformatics analysis of male mice revealed that Nanoplastics (NPs) activated inflammatory and cell death pathways in the hippocampus (HIP), with upregulation of key genes such as Selp, Timp1, and Emp3. In the medial prefrontal cortex (mPFC), NS exposure upregulated pathways associated with neuronal apoptosis, reactive oxygen species (ROS) processes, and cell death, accompanied by dysregulated expression of genes such as Tert, Adam8, Esr2, and Emp3 (69). Vitamin K2 (VK2) pretreatment rescued these molecular alterations by normalizing the expression of key genes (69). Additionally, VK2 reduced levels of IL-1β, TNF-*α*, IL-6, ROS and MDA, while attenuating brain cell apoptosis and microglial activation in the HIP and mPFC of male mice. This study demonstrates that NS exposure increases the susceptibility of young adults to perioperative neurocognitive disorders (PNDs) and highlights VK2 as a promising preventive strategy (69).

RNA sequencing results revealed a negative correlation between fibroblast growth factor 1 (FGF1) and the activation of lipophagy (70). By suppressing lipophagy, exogenous FGF1 treatment effectively blocked neuroinflammation and lipid accumulation induced by microplastics both *in vitro* and *in vivo* (70). Furthermore, exogenous FGF1 administration ameliorated learning and memory impairments as well as neuropathological damage in mice exposed to PS-NPs. These findings suggest that FGF1 may serve as a potential neuroprotective agent against PS-NPs-induced neural injury by remodeling central nervous system lipid metabolism (70). Cheng Tan et al., highlighted the role of the Piezo1/CaN/NFAT1 axis in polystyrene nanoplastics (PSNPs)-induced ER Ca2 + homeostasis imbalance, which was effectively inhibited by luteolin (LUT). Notably, LUT alleviated the susceptibility to striatal ferroptosis induced by PSNPs via the G6PD/glutathione axis (71).

## 4 Conclusion

Microplastics can enter the human body through multiple routes such as ingestion, inhalation, and dermal contact, permeating various organs including the brain, lungs, spleen, intestines, and reproductive system. This infiltration leads to diverse pathological alterations, such as neurotoxicity, inhibition of cell proliferation, and suppression of immune responses. From embryonic stages to adulthood, microplastics indiscriminately cause harm to humans, demonstrating characteristics of age-independent, non-target-organ-restricted, and multi-level pathological damage. These properties pose a severe threat to human health. The impact of microplastics on the central nervous system represents a complex and critically underexplored field of research.
